# From the lab to the field: acceptability of using electroencephalography with Indian preschool children

**DOI:** 10.12688/wellcomeopenres.17334.1

**Published:** 2022-03-18

**Authors:** Georgia Lockwood Estrin, Supriya Bhavnani, Amy Goodwin, Rashi Arora, Gauri Divan, Rianne Haartsen, Luke Mason, Vikram Patel, Mark H. Johnson, Emily J.H. Jones

**Affiliations:** 1 Centre for Brain and Cognitive Development, Department of Psychological Sciences, Birkbeck, University of London, London, WC1E 7JL, UK, UK; 2 School of Psychology, University of East London, London, E16 2RD, UK, UK; 3 Child Development Grou, Sangath, House 451 BhatkarWaddo, Succor, Bardez, Goa, 403501, India; 4 Institute of Psychology, Psychiatry and Neurosciences, King's College London SE1 1UL, London, UK; 5 Department of Global Health and Population, Harvard T H Chan School of Public Health, Boston, USA; 6 Department of Psychology, University of Cambridge, Cambridge, CB2 3EB, UK

**Keywords:** child development, EEG, low and middle income country, qualitative research, neuroethics

## Abstract

**Background**: Measurement of social and cognitive brain development using electroencephalography (EEG) offers the potential for early identification of children with elevated risk of developmental delay. However, there have been no published reports of how acceptable EEG technology is to parents and children within communities, especially in low-resource contexts such as in low and middle income countries (LMICs), which is an important question for the potential scalability of these assessments. We use a mixed-methods approach to examine whether EEG assessments are acceptable to children and their caregivers in a low resource community setting in India.

**Methods**: We assessed the acceptability of neurophysiology research and *Braintools* (a novel neurodevelopmental assessment toolkit using concurrent EEG and eye-tracking technology) using: 1) a child engagement measure, 2) interviews with caregivers (n=8); 3) survey about caregiver’s experience (n=36). Framework analysis was used to analyse interview data.

**Results**: Key topics were examined using the framework analysis: 1) parental experience of the assessment; and 2) the acceptability of research. From topic 1, four sub-themes were identified: i) caregivers’ experience of the assessment, ii) caregivers’ perception of child's experience of assessment, iii) logistical barriers and facilitators to participation, and iv) recommendations for improvement. From topic 2, three themes were identified: i) caregivers' understanding of the research, ii) barriers to participation, and iii) facilitators to participation.

**Conclusions**: We demonstrate for the first time the acceptability of conducting neurodevelopmental assessments using concurrent EEG and eye-tracking in preschool children in uncontrolled community LMIC settings. This kind of research appears to be acceptable to the community and we identify potential barriers and facilitators of this research, thus allowing for future large scale research projects to be conducted investigating neurodevelopment and risk factors for suboptimal development in LMICs.

## Introduction

Over 200 million children in low and middle income countries (LMICs) are estimated to be at risk of suboptimal brain and cognitive development, a large proportion of whom reside in India ^1^. Children growing up in LMICs experience a disproportionate burden of risk factors for suboptimal development, such as poverty ^2, 3^. Further to this, in LMICs there is also a high prevalence of neurodevelopmental disorders in children ^4^, the majority of whom are not identified and are therefore unable to access health services and intervention ^5, 6^. There is thus a global demand to develop measures to assess neurodevelopment for timely identification of children in need of intervention and health care services. Despite this, the majority of our current knowledge and understanding of brain development and associated risk factors for suboptimal development results from samples in high-income countries (HICs). There is thus an urgent need to expand our research of neurodevelopment to investigate large-scale risk factors in children beyond these settings.

Electroencephalography (EEG) provides a direct measure of brain activity that can be used for longitudinal assessment of neurodevelopment in field settings. It has been used to measure brain development for decades because of its objectivity, relatively low cost and ease of administration, and sensitivity to neural processing speed. Studies in infancy through to early childhood have identified EEG markers of brain development in key developmental domains associated with long-term mental health outcomes, such as cognition, attention and social communication; such markers have also been shown to be associated with the likelihood of occurrence of developmental disorders, such as autism spectrum disorder (ASD) and attention deficit hyperactivity disorder (ADHD) ^7– 11^. These markers can be observed before behavioural change is apparent, and may therefore be appropriate for early identification of children with elevated risk of developmental delay, which can pave the way for development of low-cost, and scalable interventions. EEG markers may also be sensitive to change brought about through interventions, and therefore are suitable for monitoring early-stage effectiveness ^12, 13^. However, neurodevelopmental EEG research to-date has been largely restricted to HICs and mainly conducted in laboratory-based settings ^14^. This restricts the potential generalisability of experimental results to the majority of the world’s population, and arguably to those most likely to benefit from these assessment methods. Therefore, we need to develop approaches that allow us to move these neuro-assessments to settings that can expand our reach, such as to community settings and households.

Early childhood is a particularly important stage at which to assess neurodevelopment, because these early years represent a highly dynamic stage of brain development, with brain plasticity and ability to adapt to environmental circumstances being at its peak (e.g. for review: 15). Early childhood is also a window in which behaviours associated with neurodevelopmental disorders may become apparent (e.g. for review: 16. This highlights the need for a greater understanding of how to identify and support young children who may be at an increased likelihood of later difficulties. Indeed, there is some evidence that differences in support during the preschool years may impact ^12, 17^; and potentially mediate ^18^ the effect of risk factors such as poverty on brain development.

Although the preschool years are a sensitive period to study developmentally ^19, 20^, studies in this age range remain limited due to the well-known difficulties of engaging young children and maintaining their attention during neurodevelopmental assessments ^21^. These challenges also affect the acceptability of conducting neurophysiological assessments for the family, the child and the community. To overcome these difficulties in capturing and maintaining child attention during tasks we developed a toolbox – *Braintools* - that uses a gaze-contingent stimulus presentation approach while measuring neural responses with a low-density, wearable, and portable EEG system. *Braintools* includes a range of visual and auditory processing task aimed to examine individual differences in early brain development. Four aspects of the toolbox were focused on maximising acceptability to children and families. First, we use a wearable and wireless EEG system that allows the child to move freely around the room; this may appear less intimidating than typical wired lab-based setups. Second, tasks are programmed to be gaze-contingent, allowing stimuli to be presented when the child is looking at the screen only. This approach automatically adapts to noisy or disruptive settings where the child might become easily distracted. Third, the gaze contingent stimulus presentation also allows the child to modulate the speed of the task to suit their own attention needs. These aspects aim to target the challenge of low data availability and high drop-out rates in developmental studies ^22^. Finally, having eye-tracking removes the need for collecting videos of the child for later manual gaze coding; this improves the potential for scalability and reduces the privacy concerns raised by capturing images of the child in their home setting. A recent study in a HIC and laboratory-based setting demonstrated this gaze-contingent stimulus presentation results in low drop-out rates and results in moderate test-retest reliability of neural responses in young toddlers ^23^.

Due to the increasing availability of relatively low-cost and portable EEG and eye-tracking devices ^5, 24^, the time is ripe to test the potential for scalability of the *Braintools* toolbox in low resource settings. However, we currently only have limited practical examples of whether EEG can be used to assess neurodevelopment in naturalistic, community-based settings, especially in low-resource contexts such as in LMICs ^25^. Indeed, there have been no published reports of how acceptable EEG technology is to parents and children within communities, which is an important question for the potential scalability of these assessments. Furthermore, there has been very little research on the overall impact of neurophysiological research in different cultural settings. As cultural values can influence how scientific advances are understood and adopted by society ^26^, it is important to understand perspectives from different settings, including LMICs.

Here, we assessed the acceptability of *Braintools* in children aged 3–5 years in low resource community settings, specifically in New Delhi, India. This study is part of a larger programme testing the reliability of EEG conducted in community settings and represents the first critical step to examine acceptability in the community of conducting *Braintools*. To do this, we first examine the child’s engagement with *Braintools* as a behavioural measure of acceptability to the child. Second, as we are working within communities previously unfamiliar with neurophysiological research, we use a mixed-methods approach to examine whether these assessments are acceptable to children and their caregivers in low resource community settings.

## Methods

### Ethics and consent

All procedures followed were approved by the local ethics committees (Institutional Ethics Committees of Sangath (approval number: GD_2018_39, dated 24/05/2018) and the Department of Psychological Sciences, Birkbeck, University of London (approval number 171897, dated 30/07/2018) and in accordance with the Helsinki Declaration of 1975, as revised in 2008.

Written informed consent for participation in the study was obtained from the parents/guardians.

### Study design and participant recruitment

Participants were purposively sampled, first through contacts that had been established during previous research projects in this area, and then by using a snowballing approach from communities to identify additional participants. The study’s objectives and methods were initially explained to potential participants by a community mobiliser at each site location. This community mobiliser was someone who was a local resident in the community where the study was being conducted, and had previously been engaged with the research team. If interested, potential participants then had the study further explained to them, and were given an opportunity to ask questions about the study with a member of the research team, prior to informed written consent being taken.

For the test phase, 40 typically-developing children aged between three and five years were recruited. Parents were asked about the mental and physical health of the children participating in the study, as well as any known history of diagnosed disorders (including developmental disorders) in the family (none were reported). Informed consent was taken from parents or caregiver of the child prior to the assessment. If the child refused to wear the EEG cap after three attempts from assessors this was taken as the child refusing to partake in the assessment. Participants were asked to complete a test and retest assessment for future examination of the reliability of brain-derived measures; acceptability was based on the initial test assessments since it is possible that the children who returned for the retest were biased towards those who found the initial protocol enjoyable. 

Data was collected over four months (from November 2018 – February 2019), and 1.5 months were spent conducting formative work.

### Study sites and set up

To assist the experimenter in introducing the elements of the *Braintools* toolbox and assessment to parents, a standard operating procedure was developed, along with verbal scripts for the local research assistant/health workers who were assisting (see Extended data). This ensured parents were fully informed about the study and technology prior to consent being taken. A driver collected participants where necessary to transport them to the testing centre.

Four community centres in Delhi/the National Capital Region (NCR) were selected. The environment of the community centres was uncontrolled, however sites were selected based on the following inclusion criteria: a) enough space to comfortably accommodate all the equipment and have place for family members accompanying the child, b) to be close to the location of families to reduce burden of travel and increase the likelihood of participation, c) sufficient illumination to enable eye-tracking calibration (see below), d) connection to a mains electricity supply.

Notes on the illumination, noise, temperature and power supply were made by the researcher at each assessment. Illumination levels varied from centres that had bright artificial lights (and where blinds were kept closed during the assessment so no natural light entered) (community centre 4), to centres where assessments relied on a small bulb and windows/open doors (community centre 1). Centres were chosen that had relatively lower background noise during testing hours. Background noise levels were measured in the noisiest of the community centres (on a busy commercial street) using a sound meter at the start, end and middle of a subset of assessments, and ranged from between 60dB-75dB. Temperature in the centres during the assessments ranged from approximately 8°Celsius during winter (no heating available), to 34°C. In all community centres there was access to a mains connection, but power cuts were frequent and therefore a portable battery was used. 

### Materials

***Parent/caregiver questionnaires and surveys.*** Parents/caregivers were asked to complete a set of questionnaires relating to family demographics, child and family medical history, which were designed to capture factors relevant to representativeness of participation. These questionnaires were translated and back-translated, and questions adapted where necessary, and then piloted with five participants. A researcher administered each question verbally with the parent/caregiver to ensure there was no difficulty with literacy skills that would affect the parent’s ability to complete the questionnaires. A copy of the questionnaires can be found in the Extended data. 

***Equipment.*** The eye-tracking equipment, used to assess child’s engagement in the task as well as to assist the automated EEG analysis, included a Tobii infra-red X2–60 attached to a monitor screen. The EEG system was chosen because of its durability in different uncontrolled climate conditions; it has also been previously implemented in LMIC settings and shown to be robust in varying environments ^25^. The EEG equipment was wireless Enobio (Neuroelectrics, Spain), 8 channels positioned at Fpz, Fz, Cz, Oz, C3, C4, P7, and P8. Data were recorded at a sampling rate of 500 Hz. The CMS and DLR electrodes were placed on an ear clip attached to the participants’ ear lobe. EEG data quality was monitored and recorded with Neuroelectrics NIC 2.0 software during the session. A portable battery (APC UPS Model: Pro – 1000) was connected to the mains supply, and all equipment was connected to the battery to receive battery power during power failures, and to avoid potential technical problems due to power surges/fluctuations during testing.

### Study procedures

***Final assessment set-up.*** The assessors included one researcher with experience working with children and EEG assessments, as well as a local research assistant/health worker. The final set up involved the child sitting on a mat on the floor in front of the monitor, which was placed on a small table of low height. The mother or caregiver typically sat next to the child and encouraged them to engage in the assessment. The assessment procedure included first placing the EEG cap on the child’s head the child, applying gel to the electrodes and testing the data quality of the EEG signal. This set-up time took between 5–20 minutes. Calibration for the eye-tracker was conducted prior to EEG tasks beginning, and involved the child sitting and watching five points on the monitor appear consecutively - calibration proceeded automatically until accuracy and precision criteria were met for at least one eye on each of five calibration points.

The complete *Braintools* battery lasted approximately 35 minutes, and included a mixture of static pictures presented when the child looked at the screen (e.g. faces, objects, checkerboards), dynamic videos (nursery rhymes sung in Hindi or toys moving – each video was approximately one-minute long) or sounds while the child either played with toys or watched a cartoon. For more information on the *Braintools* eye-tracking and EEG battery, see 23. To ensure cultural appropriateness, Hindi nursery rhymes were filmed by the research team in India, retaining properties of the video that were originally produced and used in UK research studies e.g. total number of videos, duration, colours of the video, use of gestures, etc. Participating children were familiar with the Hindi nursery rhyme used (average score for familiarity: 4.2/5).

During the assessment, if necessary, the research assistant engaged the child in the *Braintools* battery (e.g. saying names of animals/ or provided rewards of stickers after each task). Breaks were taken when and if needed by the child. Assessors took detailed notes on the assessment, including any information on child behaviour and engagement and any disruptions during assessment. For their participation, small toys were given to the child following the EEG assessment, and a kitchen storage container to parents who participated in in-depth interviews.

### Assessing the acceptability of Braintools to children

We report on practical challenges observed by the assessors on the data collection ( Table 2). To assess the acceptability of *Braintools* to children, we measured child engagement during the visual tasks using eye-tracking data. We assessed the proportion of static images where the child’s gaze was detected on the screen by the eye-tracking for greater than 30% of the time. We focus on the static images, as this took the majority of time in the assessment (approximately 90% of total time). The static stimuli were also less engaging to the children compared to the dynamic videos due to their repetitive nature, meaning that it was the task where we expect to see the greatest differences in engagement. 

### Assessing the acceptability of the research and Braintools to parents

We assessed the acceptability of neurophysiology research and the *Braintools* toolbox using two methodologies: 1) interviews with caregivers (n=8); 2)a survey about caregiver’s experience of the assessment immediately post-assessment for each participant (n=36).

Eight semi-structured in-depth interviews (IDIs) with caregivers (mothers or grandmothers) were conducted, in Hindi, to assess the acceptability of the toolbox to parents and their children. These were held approximately one month post-assessment, and in the participant’s home or community centre near to their home. Interview participants were purposefully sampled for maximally deviant participants (those whose children actively engaged during assessment vs those who did not). The topic guide included questions relating to the acceptability of the toolbox, specifically: parental expectations of the visit; experience of the visit; observation of their children’s behaviour and mood during tasks and repeat visits; understanding and expectations of the research; perceived barriers and facilitators of scaling up this research. Interviews ranged from 30 min to one-hour in duration. The topic guide can be found in the Extended data. The interviews were conducted by a female research assistant (author RA) with masters level qualifications in psychology.

For the survey, each parent was asked to score between 1 and 5 on a variety of questions about their satisfaction of the assessment (score 0 representing low levels of satisfaction; score 5 representing high levels of satisfaction). All quantitative data entry was conducted by the research assistant and double checked; a proportion (10%) was then checked again for accuracy by supervisors. 

***Data management and security.*** All digital data were transferred securely for long-term storage on a secure, encrypted server. Identifiable data was only available for researchers on this project. All quantitative and qualitative data was de-identified and anonymised for this manuscript (see Data Availability section below).

***Qualitative data processing and analysis.*** Interviews were first transcribed verbatim and translated from Hindi to English for analysis. Bilingual researchers checked for accuracy of transcription and translation. Transcripts were analysed using framework analysis, which has been widely used in health research, and especially to identify drivers and barriers to healthcare services ^27^. GLE and SB conducted coding using NVivo 12 software (alternatively, the processing and analysis can be done manually using spreadsheets). Following immersion in the interview transcripts, an initial codebook was developed. During a second immersion of the interviews, the codebook was further refined through discussion, and themes were identified to form a finalised codebook, which was applied to the remaining interviews. Any divergences of coding between authors were discussed until consensus was reached.

## Results

### Participant characteristics

Forty children took part in the study; participant characteristics are summarised in Table 1a. Children had an average age of 47.98 (± 12.23) months. It is worthy of note that for two children the precise date of birth was not known, and so an estimate was given by each parent, in the case of a discrepancy between parents, the oldest age was taken. For the majority of participants, the mother was the child’s primary caregiver (see details of participants in Table 1a). Maternal (27.8%) and paternal (38.9%) grandmothers were also key caregivers for children participating in the study. For the majority of parents (both mother and father) the highest level of education was attendance at secondary school ( Table 1b). Maternal education levels are comparible to National Family Health survey data for South Delhi ^28^.

**Table 1a.** Participant characteristics and socio-demographics.

Family Characteristics	Mean (SD)
Child age (months)	47.98 (12.23)
Maternal age (years)	24.93 (4.30)
Paternal age (years)	28.62 (5.29)
Family size (number)	6.88 (3.07)

**Table 1b.** Participant characteristics and socio-demographics.

Family Demographics			Number (proportion %) (n=40)
Child	Child characteristics	Child gender, male	23 (57.5%)
		School/nursery attendance	31 (77.5%)
	Child languages spoken	1 language spoken	17 (42.5%)
		2 languages spoken	18 (45%)
		3 languages spoken	5 (12.5%)
Mother	Maternal education (highest level attended)	none	4 (10%)
		primary	8 (20%)
		secondary	20 (50%)
		diploma	2 (5%)
		tertiary (undergraduate)	4 (10%)
		tertiary (postgraduate)	2 (5%)
	Mother employed		10 (25%)
Father	Paternal education (highest level attended)	none	1 (2.5%)
		primary	5 (12.5%)
		secondary	25 (62.5%)
		diploma	2 (5%)
		tertiary (undergraduate)	6 (15%)
		tertiary (postgraduate)	1 (2.5%)
	Father Number employed *(n=38)		35 (92.1%)

All participants were reported to be in good health, but two children had experienced a seizure once in their lifetime, one child had a parent-reported “hole in heart”, and one child’s parent reported a prior “infection in blood”.

### Data collection in the community

Of the forty families that consented to participate in the study, four (10%) children refused to wear the EEG cap, and data was therefore not collected in these cases. For three sessions, data collection was terminated before the end of the session due to technical difficulties (N = 2; software crashed and for one session the EEG data were saved incorrectly) and removal of the EEG cap by the child during data collection (N = 1). Of the 36 families who took part, 100% returned for a repeat assessment between 1–3 weeks later.

***Challenges in data collection.*** A number of challenges were faced during data collection (see Table 2), including child’s behaviour, such as inattention to the EEG tasks presented, as well as dislike of the cap and the reference ear-clip. Testing in a community setting also meant that there were multiple external distractions experienced during the assessment, such as noise from the street and vehicles, as well as disruptions from family members, especially when other children were present during the assessment. Additional challenges were faced due to power cuts and fluctuating power supply.

**Table 2.** Frequency and type of challenges observed during the assessment.

Challenges	Observations during assessment	Frequency (proportion %) (n=36)
Prior to assessment	Child dislike of the cap	2 (5.6%)
Technical and electrical	Electrical and power disruptions	2 (5.6%)
	Technology disruptions	5 (13.9%)
Child behaviour during assessment	Child excessive movement	16 (44.4%)
	Child fiddling cap or reference ear clip	13 (36.1%)
	Child became bored of visual tasks	13 (36.1%)
	Break required	5 (13.9%)
	Child terminated assessment early	5 (13.9%)
External disruptions	Background noise levels (e.g. from vehicles and road)	11 (30.6%)
	Disruptions from other family members in the room	12 (33.3%)

### Acceptability of Braintools to children

Despite challenges during data collection, we show a high level of child engagement in tasks: for static pictures, children were engaged (as defined by the child’s gaze on the screen) on average in 85.4% of trials (±12.06%; range 52.78% - 98.61%) (i.e. children engaged in an average of 241.17 (±47.96) out of a mean of 271.15 (±51.89) static pictures presented).

Two reviewers (GLE, AG) independently coded the “external disruptions” (see Table 2) experienced during the assessment. Coding was analysed based on detailed notes taken by assessors at the time of the assessment. Assessments were coded between 0 and 2; seven assessments were coded 2 (high level of external disruptions and background noise severity), ten were coded 1 (e.g some external disruptions or background noise), and the remainder (n=17) were coded 0 (no external disruptions). An ANOVA assessed the effect of external disruption on child attention to the screen during static images, using the average proportion looking time on static pictures (see details above) as the dependent variable, and external disruption coding as the independent variable. No significant effect was observed (F(2, 31) = .11, p = .90).

### Acceptability to caregivers

We assessed the acceptability of the research and *Braintools* toolbox using IDIs and a parent-report survey on their experience.

***Survey on parental experience.*** 92% (33/36) of parents reported high satisfaction of the testing methods (i.e. EEG and eye-tracking) used, and 77% (27/35) reported high overall satisfaction with the assessment visit as a whole. Parents also reported being satisfied overall with the location 83% (30/36) and duration 80% (28/35) of the assessment. In the survey, parents reported high levels of understanding of the aims of the research (91% (32/35)) and of its importance and potential for impact (83% (29/35)). Results from the survey filled out by parents immediately following the assessment were consistent with reports from the IDIs.

***Interviews.*** Key themes were identified from the framework analysis of the IDIs conducted with parents/caregivers, and these were categorised into two topics: 1) parental experience of the assessment; and 2) the acceptability of research.

Topic 1: Parental experience of the assessment

Four themes were identified from the interview transcripts on parental experience of the assessment ( Table 3), and elaborated below: 1) caregivers experience of the assessment, 2) caregivers’ perception of child's experience of assessment, 3) logistical barriers and facilitators to participation, and 4) recommendations for improvement.

**Table 3.** Experience of assessment: Themes and quotes from in-depth interviews with caregivers.

Theme	Sub-theme	Relevant quotes
**Caregivers experience of** **assessment**	Overall experience of assessment	"I liked it, that is all I would like to tell, if you would ask me to be a part of it again then I would definitely be willing to be a part of this."
		"I liked everything about it."
	Comparison to other child assessments	"If I go to the doctor, this was a study of the brain of the child, it was better than that, children would get tensed, irritated and fidgety but the child was sitting peacefully through the child’s brain test."
**Caregiver's perception of child's ** **experience of assessment**	Overall enjoyment of assessment	"The child was just happy to be a part of this, she felt like it part of some game that she was enjoying."
		"The child did really well, the things the child doesn’t agree to at home, he did it here."
	Child exceeding parental expectations	"My child doesn’t like wearing cap but the child wore the cap for half an hour and sat through the test."
		"I was hoping that my child would sit through it, I didn’t think my child would and it felt really nice when the child did."
	Challenges in assessment	"The child got a little fussy about washing the gel, she wanted me to get it off."
		"I was sitting behind the child as she was turning again and again that if I am around or not."
**Barriers and facilitators to** ** participation**	Logistical barriers to participation	"I also said that I don’t have time to participate in this, where I go and sit in the centre for 2-3 hours. I have a lot of work at house, sometimes I don’t get time to have food till 2 p.m."
		"If someone has younger kids or they don’t have time then it would be really difficult for them."
		"Maybe there are families with both parents working so they might not have time to participate. The children might also be going to school."
		"You asked to come directly to that place, I couldn’t as I didn’t know the way, I don’t usually go out of the house."
	Logistical facilitators - transport	"You took me through car so I was able to reach there or else if I had to come all by myself then it would have been difficult and I couldn’t have been able to reach there."
	Logistical facilitators - proximity of testing centre	"That’s why I confirmed if we would have to come outside [the community] somewhere as it might be impossible for me to do so but then the girl told me that the assessors would come over and do it."
**Recommendations for** ** improvement**	Location	"This room where test is done is too simple if there is something like designs to interest them or there are toys of animals to play with then the children would be attracted towards it more."
		"Everybody doesn’t have time that they come over and participate so it would be better if you go over to their house and do it there."
	Assessment and stimuli	"If you would add more cartons then the children would enjoy it more."
		"Like if you are evaluating a child’s brain but if you also include evaluating the child physically then more people might be willing to be a part of this."

*Theme 1: caregivers experience of the assessment*

Overall, mothers reported enjoying the assessment and were happy to have their child take part.

"I liked it, that is all I would like to tell, if you would ask me to be a part of it again then I would definitely be willing to be a part of this." – *Mother*

Mothers reported initial concerns regarding the assessment; the majority of these concerns were experienced prior to the assessment itself, and related to the use of the plastic tube required to apply the EEG gel for the assessment, the cap, and the gel itself. Mothers also reported concern that their child would not comply with sitting still throughout the assessment. However, after speaking with assessors and seeing equipment, their concerns diminished:

“I was confused as to how will it be used but then when you did the test then I came to know.” *– Mother*

Mothers also reported that researchers’ engagement of children in playing games during the assessment were helpful for reducing their concerns. Caregivers compared the assessment to their experiences of a doctor’s visit, describing their preference for the EEG assessment.

*Theme 2: caregivers’ perception of child's experience of assessment*

Many positive comments from caregivers emerged when asked about their child’s response to the assessment. This enjoyment was mainly described by parents of children who were attentive during the assessment, but was also mentioned by parents of those who were difficult to engage.

“The child was just happy to be a part of this, she felt like it part of some game that she was enjoying." – *Mother*

Mothers also reported their child exceeding expectations of engagement with the task and of wearing the cap, which demonstrates the acceptability of the assessment to the child.

"I was hoping that my child would sit through it, I didn’t think my child would and it felt really nice when the child did." – *Mother*

Parents of children who did not engage throughout the assessment mentioned that their child disliked the cap and the gel.

"My child doesn’t like wearing cap but the child wore the cap for half an hour and sat through the test." – *Mother*

*Theme 3: logistical barriers and facilitators to participation*

Caregivers reported logistical barriers to participation, specifically focusing on the duration of the assessment, difficulties of leaving the home to participate, and challenges to the mother of having to balance working in the home or their job with taking their child to the assessment.

"Maybe there are families with both parents working so they might not have time to participate. The children might also be going to school." – *Mother*
"You asked to come directly to that place, I couldn’t as I didn’t know the way, I don’t usually go out of the house." – *Mother*

A key facilitator to participation was providing the assessment centre close to participant’s homes, and providing a means of transport if required to and from the assessment.

"That’s why I confirmed if we would have to come outside [the community] somewhere as it might be impossible for me to do so but then the girl told me that the assessors would come over and do it." – *Mother*

*Theme 4: recommendations for improvement*

The final theme identified recommendations for improvement of the assessment by parents, mentioning the importance of assessments being in close proximity to their home, and how parents enjoyed the videos compared to static images part of the assessment (see Table 3).

"Everybody doesn’t have time that they come over and participate so it would be better if you go over to their house and do it there." *- Mother*

Topic 2: Acceptability of research

Three themes were identified from the interview transcripts on the acceptability of research (see Table 4): 1) caregivers' understanding of the research, 2) barriers to participation, and 3) facilitators to participation.

**Table 4.** Acceptability of research: Themes and quotes from in-depth interviews with caregivers.

Theme	Sub-theme	Relevant quotes
**Caregivers ** **understanding ** **of the research**	Understanding of motivation of research	"She [project personnel] told me that you are trying to understand the difference between the normal children and children with some difficulties."
		"Sometimes our children have some kinds of troubles which needs to be identified at an earlier stage so that it can be taken care of accordingly, so this was the test to identify that."
		I was thinking that why would you test my child’s brain as she is absolutely fine.
	Misconceptions about research	"If there is something wrong with the child, you told that you would tell us and also if there is something wrong then you would also take care of the child."
		I would become aware about the child’s future.
	Concern about equipment having an adverse impact on child's brain	"I was just a little worried about the machines that would be attached to the child’s head, how it would affect the child’s brain and the child itself ."
		"I just didn’t understand about the headpiece, everything else was fine."
		"They said that they would be making the child wear a cap so I asked what kind of cap, then they told us that they would be applying a gel and then making the child wear a cap so I was worried if the child’s brain would be affected by it."
**Barriers to ** **participation**	Family members' objections	"I told him [child's father] that the child would be asked to wear a cap and the child’s brain would be seen in a computer so he refused to make the child wear the cap as it might affect the child’s brain."
	Perceived harm of assessment by community	"A few people in my locality didn’t agree to get this test done as they thought that it would affect their child’s cognition abilities."
**Facilitators to** ** participation**	Family members' opinion	"They told mummy [paternal grandmother] about it, mummy told me and then she said that nothing bad will happen, you may get it done."
		"My husband agreed so I did."
		"My father was really excited about it as nobody in my family knew about it so when I told them about
		it, they really liked it."
		Then mummy said that everybody is getting it done so we should also get it done.
	Community mobiliser's encouragement	"She [community mobiliser] is well read and has knowledge about these things so if she is saying then I agreed to get it done."
		"I asked if anything would happen to my girl and she [community member] said no nothing will happen."
	Other's participation of assessment	"When other people are participating then you can also, nothing bad would happen then. It is about children, about their thinking, about their future, that is it."
		"My sister had participated in this so she said it is okay to do it."
	Confidence in own understanding of assessment	"We are smart enough to understand what is right and wrong for us, we wouldn’t have said yes if we wouldn’t have found it right. It is not like that if we have to agree to everything they say, if it is one then we would say it is one and wouldn’t call it two."
	Benefit to society	"I don’t have much knowledge, all I want is that other children would be benefitted by my child’s participation so I agreed to it, even though it would not affect my child’s cognition."
		"I agreed to it as I didn’t find anything wrong about it, you are doing this for the children, you are doing this for the benefit of the children."

*Theme 1: caregivers' understanding of the research*

The first theme highlighted the breadth of different perspectives and understanding of research by parents participating in this study. Some mothers described the research as focusing on assessment of *“normal children and children with some difficulties”*, others highlighted that it was beneficial for early identification of delays in development.

"Sometimes our children have some kinds of troubles which needs to be identified at an earlier stage so that it can be taken care of accordingly, so this was the test to identify that." - *Mother*

However, some mothers reported that they had expected results or reports to be provided about their child’s development. This was mentioned in both interviews and the survey. Other parents highlighted initial concern about the equipment having an adverse impact on their child's brain. However, parent’s concerns were alleviated upon seeing the equipment and speaking with the research team.

"They said that they would be making the child wear a cap so I asked what kind of cap, then they told us that they would be applying a gel and then making the child wear a cap so I was worried if the child’s brain would be affected by it." – *Mother*
"I was just a little worried about the machines that would be attached to the child’s head, how it would affect the child’s brain and the child itself." – *Mother*

*Theme 2: barriers to participation*

Parents reported potential barriers to participation in research. A key barrier mentioned was the perception and concern about the assessment by family and community members, specifically any perceived harm of the assessment.

"A few people in my locality didn’t agree to get this test done as they thought that it would affect their child’s cognition abilities." – *Mother*

One mother also mentioned that testing children away from the home raised concerns with some community members regarding the safety of the child.

*Theme 3: facilitators to participation*

Mothers also described facilitators to participation in research, and in many cases their husband’s agreement was the biggest facilitator to participation.

"My husband agreed so I did." – *Mother*

Other facilitators identified included the excitement about the study from other family members.

“Then mummy [paternal grandmother] said that everybody is getting it done so we should also get it done.” – *Mother*

The results from these interviews highlighted the importance of engaging the community with research, as well as the importance of the community mobiliser in encouraging families to take part.

"She [community mobiliser] is well read and has knowledge about these things so if she is saying then I agreed to get it done." – *Mother*

## Discussion

This study aimed to assess the acceptability of concurrent EEG and eye-tracking data capture to families in LMIC community settings, and to identify barriers for future implementation of neurodevelopmental assessments. The majority of developmental EEG research has been conducted in HIC. Despite previous projects demonstrating successful EEG data acquisition to assess infant and child development in LMICs, including in Malawi ^29^, The Gambia ^30^ and Bangladesh ^31^, there remains a scarcity of literature detailing the cultural acceptability of these methods to study neurodevelopment in LMICs. These projects have been based in laboratory or hospital settings; but there remain unique additional challenges to conducting EEG testing in community LMIC settings that have not previously been fully investigated.

To assess the acceptability of these assessments to children, we measured their engagement with a visual battery presented while EEG and eye-tracking was concurrently captured. We found that children were attentive and engaged with the visual stimuli presented. One of the major barriers of concern in testing outside a lab-setting is noise levels, and interestingly, we found that external disruptions and noise did not correlate with children’s engagement with the tasks. This may be because tests were conducted in community settings, close to the children’s home, and often on the same street where the child lived; these noises and disruptions may therefore be typical for the child and not be distracting. Comparatively, it could be argued that a lab-based set up, despite displaying a controlled environment with no disruptions to the assessment, is an unfamiliar and unnatural environment for the child, and at minimal cost to data quality and quantity, the gaze-controlled EEG paradigm can be moved to more naturalistic and ecologically valid settings. 

### Acceptability of neurophysiological research

We sought to investigate whether communities are comfortable with neurophysiological research being conducted in LMIC settings, and we demonstrate here that gaze-controlled EEG research was acceptable to both children and their parents in the communities we were working with in New Delhi. We found, both from parental survey data collected immediately following the assessment, as well as from in-depth interviews with a smaller number of parents, that parental experience of the assessment was overall positive, and often exceeded expectations. Interestingly, in this small population we found mixed levels of understanding of the research, as well as of the benefits vs risks of research. Whilst the survey suggested high levels of understanding about the research, the more in-depth approach via interviews revealed some misunderstanding. Interviews with parents highlighted that whilst parents generally thought this research was important “ *for the benefit of the children”,* some parents had misconceptions about the individual benefits they would receive, for example an expectation of detailed information about their child’s development, e.g. as one parent stated, “ *I would become aware about the child’s future”*. Despite careful consideration being taken to highlight that individual feedback would not be possible, this issue was raised in more than one case. Informed consent had included provision of a written information sheet explicitly stating that individual feedback was not possible, and researchers emphasised this at both the start and end of the face-to-face discussion with parents about the study, prior to consent being taken. This therefore highlights that ethical and practical guidelines are needed to help researchers reduce potential misconceptions of participation benefits. Such ethical considerations of the process of consent have been previously emphasised surrounding using neuroimaging techniques in HICs, emphasising the need for additional considerations in LMICs ^32^. Our study therefore highlights key questions, challenges, barriers and facilitators regarding the implementation and neuro-ethical implications of this work.

There has been little research conducted to-date on understanding the implications of conducting neuroscientific work in different cultural settings ^26^. This study is a step to bringing some understanding to this field, and it highlights that continued explorations of the acceptability of field-based neuroimaging research needs to be conducted. A key theme that emerged from the interviews were parental concerns over potential harms of the assessment to their child and how the equipment “ *would be attached to the child’s head, how it would affect the child’s brain and the child itself ."* However, parents generally noted that any concerns they had were relieved upon speaking with researchers or seeing the equipment. Parental and child’s overall acceptance of the assessment is evidenced by the observation that 100% of parents and children who took part in the assessment, agreed and came back for a second visit approximately one week later. We also specifically asked parents about other members of their community and their views on the assessments being conducted. Our findings emphasised the importance of creating community buy-in about research, and also engaging community members to ensure that any misconception of research was not a reason for refusal to participate; for example it was reported by one parent that “ *a few people in my locality didn’t agree to get this test done as they thought that it would affect their child’s cognition abilities*.”

Agreement of husbands, parents in law and other community members were key to mother’s agreement to participate in research, thereby further highlighting how important it was to create community buy-in, and their involvement in the consent protocol and in recruitment strategies. Such “collective culture” has often been described in comparison to more “individualistic” Western cultures, and this extends to healthcare decision making within families ^26^, where it may be common practice for the head of the family to make such decisions ^33^. Similar findings highlighting the importance of engaging community and other family members have also been found when studying health services for child development in other LMICs e.g. Thailand ^34^. 

### Limitations

A limitation of our study was that our recruitment strategy was not random, instead involving purposive sampling and through contacts within the community through participation in previous child development research projects, thereby making it impossible to determine future participation rates for population-based cohorts. However, our cohort had comparable demographic data (e.g. maternal education level) to that of Family Health Survey data for South Delhi, suggesting the sample was fairly representative of the local area. We also interviewed only parents who agreed to participate in the study, and therefore, have not captured the first-hand perspectives of parents who may have had other reservations. A larger study, across multiple cultural contexts is therefore warranted to further establish the acceptability as well as the evidence of utility of this gaze-controlled EEG paradigm to assess early brain development in multiple cultural contexts. A further limitation of this study is that the assessment was administered by experienced EEG researchers, and therefore subsequent studies need to build evidence that non-specialists can be trained to conduct the assessment. Once such validation studies have been conducted, toolboxes such as these will have potential to benefit children and families from LMIC, by providing improved access for detection of developmental disorders and early referral to intervention and care. This technology offers the additional advantage of being able to measure change brought about through interventions and therefore could be a valuable tool in future trials to measure effectiveness of early intervention ^13^. A key advantage of the *Braintools* toolbox is the potential for it to be administered by non-experts and health care workers within the community with minimal training and therefore its applicability for scalable healthcare solutions ^5^.

## Conclusion

In this paper, we demonstrate for the first time the acceptability of conducting neurodevelopmental assessments using the *Braintools* toolbox in preschool children in uncontrolled community LMIC settings. We show that despite challenges including excessive noise disruptions, children were effectively engaged in the assessments to allow for collecting data using simultaneous EEG and eye-tracking technologies. Importantly, we demonstrate that this kind of research appears to be acceptable to the community, allowing for future large scale research projects to be conducted investigating neurodevelopment and risk-factors for suboptimal development in LMICs.

## Data availability

### Underlying data

Birkbeck Research Data Repository (BiRD): BrainTools Implementation. https://doi.org/10.18743/DATA.00183 ^35^

This project contains the following underlying data:

- BrainToolsDemographicData.docx (individual-level demographic data)
- BrainToolsFeasibilityData.docx (individual-level feasibility data - including notes taken by observers)
- BrainToolsAcceptabilityData.docx (individual-level acceptability data)

Data are available under the terms of the Creative Commons Attribution 4.0 International license (CC-BY 4.0).

This manuscript contains qualitative data that cannot be made available to the public, as data consists of transcriptions of in-depth interviews that cannot be effectively de-identified and free answers to the questionnaire that include ethnicity of the participants, and therefore the datasets must not be shared in order to protect patient/participant privacy. For more information about access to this data, please contact the corresponding author of this manuscript; we will only provide the datasets to researchers who become associated with this project and provide detailed proposals of how this work will be shared and analysed.

### Extended data

Extended data can be found: Birkbeck Research Data Repository (BiRD): BrainTools Implementation. https://doi.org/10.18743/DATA.00183 ^35^

Extended data includes:

- Verbal script for Braintools assessment procedure.docx (verbal scripts for health workers conducting assessment)
- Braintools Topic guide (Topic guide for interviews (English))
- Parent feedback form_English.docx (parent feedback form for acceptability data)
- Socio-Demographics form_English.docx (socio-demographic questionnaires)

Data are available under the terms of the Creative Commons Attribution 4.0 International license (CC-BY 4.0).

## References

[1] Lu C Black MM Richter LM : Risk of poor development in young children in low-income and middle-income countries: an estimation and analysis at the global, regional, and country level. *Lancet Glob Health.* 2016;4(12):e916–22. 10.1016/S2214-109X(16)30266-2 27717632 PMC5881401

[2] Black MM Walker SP Fernald LCH : Early childhood development coming of age: science through the life course. *Lancet.* 2017;389(10064):77–90. 10.1016/S0140-6736(16)31389-7 27717614 PMC5884058

[3] Britto PR Lye SJ Proulx K : Nurturing care: promoting early childhood development. *Lancet.* 2017;389(10064):91–102. 10.1016/S0140-6736(16)31390-3 27717615

[4] Arora NK Nair MKC Gulati S : Neurodevelopmental disorders in children aged 2– 9 years: Population-based burden estimates across five regions in India.Persson LÅ, editor. *PLOS Med.* 2018;15(7):e1002615. 10.1371/journal.pmed.1002615 30040859 PMC6057634

[5] Dasgupta J Bhavnani S Estrin GL : Translating neuroscience to the front lines: point-of-care detection of neuropsychiatric disorders. *Lancet Psychiatry.* 2016;3(10):915–7. 10.1016/S2215-0366(16)30186-9 27692259

[6] Durkin MS Elsabbagh M Barbaro J : Autism screening and diagnosis in low resource settings: Challenges and opportunities to enhance research and services worldwide. *Autism Res.* 2015;8(5):473–6. 10.1002/aur.1575 26437907 PMC4901137

[7] Gabard-Durnam LJ Wilkinson C Kapur K : Longitudinal EEG power in the first postnatal year differentiates autism outcomes. *Nat Commun.* 2019;10(1):4188. 10.1038/s41467-019-12202-9 31519897 PMC6744476

[8] The BASIS Team, Orekhova EV Elsabbagh M : EEG hyper-connectivity in high-risk infants is associated with later autism. *J Neurodev Disord.* 2014;6(1):40. 10.1186/1866-1955-6-40 25400705 PMC4232695

[9] The BASIS team, Haartsen R Jones EJH : Functional EEG connectivity in infants associates with later restricted and repetitive behaviours in autism; a replication study. *Transl Psychiatry.* 2019;9(1):66. 10.1038/s41398-019-0380-2 30718487 PMC6361892

[10] Jones EJH Venema K Earl R : Reduced engagement with social stimuli in 6-month-old infants with later autism spectrum disorder: a longitudinal prospective study of infants at high familial risk. *J Neurodev Disord.* 2016;8(1):7. 10.1186/s11689-016-9139-8 26981158 PMC4791854

[11] Dickinson A DiStefano C Senturk D : Peak alpha frequency is a neural marker of cognitive function across the autism spectrum. *Eur J Neurosci.* 2018;47(6):643–51. 10.1111/ejn.13645 28700096 PMC5766439

[12] Dawson G Jones EJH Merkle K : Early Behavioral Intervention Is Associated With Normalized Brain Activity in Young Children With Autism. *J Am Acad Child Adolesc Psychiatry.* 2012;51(11):1150–9. 10.1016/j.jaac.2012.08.018 23101741 PMC3607427

[13] Jones EJH Dawson G Kelly J : Parent-delivered early intervention in infants at risk for ASD: Effects on electrophysiological and habituation measures of social attention.Intervention in infants at risk for ASD. *Autism Res.* 2017;10(5):961–72. 10.1002/aur.1754 28244271 PMC5993545

[14] Bhavnani S Lockwood Estrin G Haartsen R : EEG signatures of cognitive and social development of preschool children-a systematic review. *PloS One.* 2021;16(2):e0247223. 10.1371/journal.pone.0247223 33606804 PMC7895403

[15] Lockwood Estrin G Bhavnani S : Brain Development: Structure.In: *Encyclopedia of Infant and Early Childhood Development.* Elsevier; 2020 [cited 2021 Feb 22];205–14.

[16] Zwaigenbaum L Bryson S Garon N : Early identification of autism spectrum disorders. *Behav Brain Res.* 2013;251:133–46. 10.1016/j.bbr.2013.04.004 23588272

[17] Kok R Thijssen S Bakermans-Kranenburg MJ : Normal Variation in Early Parental Sensitivity Predicts Child Structural Brain Development. *J Am Acad Child Adolesc Psychiatry.* 2015;54(10):824–831.e1. 10.1016/j.jaac.2015.07.009 26407492

[18] Luby J Belden A Botteron K : The effects of poverty on childhood brain development: the mediating effect of caregiving and stressful life events. *JAMA Pediatr.* 2013;167(12):1135–42. 10.1001/jamapediatrics.2013.3139 24165922 PMC4001721

[19] Guyer AE Pérez-Edgar K Crone EA : Opportunities for Neurodevelopmental Plasticity From Infancy Through Early Adulthood. *Child Dev.* 2018;89(3):687–97. 10.1111/cdev.13073 29664997 PMC5948168

[20] Luby JL Belden A Harms MP : Preschool is a sensitive period for the influence of maternal support on the trajectory of hippocampal development. *Proc Natl Acad Sci U S A.* 2016;113(20):5742–7. 10.1073/pnas.1601443113 27114522 PMC4878487

[21] Bell MA Cuevas K : Using EEG to Study Cognitive Development: Issues and Practices. *J Cogn Dev.* 2012;13(3):281–94. 10.1080/15248372.2012.691143 23144592 PMC3491357

[22] Noreika V Georgieva S Wass S : 14 challenges and their solutions for conducting social neuroscience and longitudinal EEG research with infants. *Infant Behav Dev.* 2020;58:101393. 10.1016/j.infbeh.2019.101393 31830682

[23] Haartsen R Mason L Braithwaite E : Reliability of an automated gaze-controlled paradigm for capturing neural responses during visual and face processing in toddlerhood. *Dev Psychobiol.* 2021;63(7):e22157. 10.1002/dev.22157 34674242 PMC9293026

[24] Lau-Zhu A Lau MPH McLoughlin G : Mobile EEG in research on neurodevelopmental disorders: Opportunities and challenges. *Dev Cogn Neurosci.* 2019;36:100635. 10.1016/j.dcn.2019.100635 30877927 PMC6534774

[25] Katus L Hayes NJ Mason L : Implementing neuroimaging and eye tracking methods to assess neurocognitive development of young infants in low- and middle-income countries [version 2; peer review: 2 approved]. *Gates Open Res.* 2019;3:1113. 10.12688/gatesopenres.12951.2 31508580 PMC6719506

[26] Rommelfanger KS Jeong SJ Ema A : Neuroethics Questions to Guide Ethical Research in the International Brain Initiatives. *Neuron.* 2018;100(1):19–36. 10.1016/j.neuron.2018.09.021 30308169

[27] Gale NK Heath G Cameron E : Using the framework method for the analysis of qualitative data in multi-disciplinary health research. *BMC Med Res Methodol.* 2013;13:117. 10.1186/1471-2288-13-117 24047204 PMC3848812

[28] National Family Health Survey - 4. Ministry of Health and Family Welfare, Government of India;2016. Reference Source

[29] Leal Neto O Haenni S Phuka J : Using wearable devices and mobile surveys for child and youth development in Malawi: An implementation study of high-frequency data collection based in multi-modal approach. *JMIR Public Health Surveill.* 2021. Reference Source 10.2196/23154 PMC7980111 33536159

[30] Katus L Mason L Milosavljevic B : ERP markers are associated with neurodevelopmental outcomes in 1-5 month old infants in rural Africa and the UK. *NeuroImage.* 2020;210:116591. 10.1016/j.neuroimage.2020.116591 32007497 PMC7068721

[31] Jensen SKG Kumar S Xie W : Neural correlates of early adversity among Bangladeshi infants. *Sci Rep.* 2019;9(1):3507. 10.1038/s41598-019-39242-x 30837491 PMC6401115

[32] Shen FX Wolf SM Bhavnani S : Emerging Ethical Issues Raised by Highly Portable MRI Research in Remote and Resource-Limited International Settings. *NeuroImage.* 2021;238:118210. 10.1016/j.neuroimage.2021.118210 34062266 PMC8382487

[33] Tai MCT Lin CS : Developing a culturally relevant bioethics for Asian people. *J Med Ethics.* 2001;27(1):51–4. 10.1136/jme.27.1.51 11233380 PMC1733344

[34] Morrison J Chunsuwan I Bunnag P : Thailand’s national universal developmental screening programme for young children: action research for improved follow-up. *BMJ Glob Health.* 2018;3(1):e000589. 10.1136/bmjgh-2017-000589 29564160 PMC5859813

[35] Estrin L Georgia Bhavnani S : BrainTools Implementation. Birkbeck College, University of London.2021. 10.18743/DATA.00183

