# Supplementary material for: From the lab to the field: acceptability of using electroencephalography with Indian preschool children
Source: Wellcome Open Res. 2023 Oct 12;7:99. Originally published 2022 Mar 18. [Version 2] doi: 10.12688/wellcomeopenres.17334.2 (PMC10632594; doi:10.12688/wellcomeopenres.17334.2)
Supplement: Peer review history

# Reviewer report: Reviewer response for version 2

**DOI:** 10.21956/wellcomeopenres.22380.r68570

**Published:** 2023-11-08

**Authors:** Nicholas Badcock

**Affiliations:** 1 School of Psychological Sciences, University of Western Australia, Perth, WA, Australia

Dear Georgia and colleagues,

 Thank for your considered responses to these enquiries. I'm happy with the responses and I think the additional figure in response to Reviwer 1 is a nice inclusion.

 I have no further comments.

 Best wishes,

 Nic

Is the work clearly and accurately presented and does it cite the current literature?

Yes

If applicable, is the statistical analysis and its interpretation appropriate?

Yes

Are all the source data underlying the results available to ensure full reproducibility?

Yes

Is the study design appropriate and is the work technically sound?

Yes

Are the conclusions drawn adequately supported by the results?

Yes

Are sufficient details of methods and analysis provided to allow replication by others?

Yes

Reviewer Expertise:

I'm a cognitive neuroscientist with a background in psychology and quantitative methods. I have some experience with qualitative methods.

I confirm that I have read this submission and believe that I have an appropriate level of expertise to confirm that it is of an acceptable scientific standard.

# Reviewer report: Reviewer response for version 2

**DOI:** 10.21956/wellcomeopenres.22380.r68569

**Published:** 2023-10-19

**Authors:** Wanze Xie

**Affiliations:** 1 School of Psychological and Cognitive Sciences, Peking University, Beijing, China

The authors have addressed my questions in the lastest version of the manuscript.  I have no further comments to make.

Is the work clearly and accurately presented and does it cite the current literature?

Yes

If applicable, is the statistical analysis and its interpretation appropriate?

Yes

Are all the source data underlying the results available to ensure full reproducibility?

Partly

Is the study design appropriate and is the work technically sound?

Yes

Are the conclusions drawn adequately supported by the results?

Yes

Are sufficient details of methods and analysis provided to allow replication by others?

Partly

Reviewer Expertise:

EEG/ERP, child development

I confirm that I have read this submission and believe that I have an appropriate level of expertise to confirm that it is of an acceptable scientific standard.

# Reviewer report: Reviewer response for version 1

**DOI:** 10.21956/wellcomeopenres.19164.r52059

**Published:** 2022-10-25

**Authors:** Nicholas Badcock

**Affiliations:** 1 School of Psychological Sciences, University of Western Australia, Perth, WA, Australia

**Overview:**

 The paper examines the acceptability of Braintools (combined eye-track and EEG for cognitive testing) in a low and middle income country (LMIC) using a mixed methods design. They examine data interference due to noise in non-laboratory environments and acceptability through survey and interviews. The report is positive with respect to the tools and techniques employed.

 Dear Dr Lockwood-Estrin and colleagues,

 Very nice work! I enjoyed reading this and am increasingly excited about the potential of this space. Well done on making it happen – feels like a massive logistic challenge. Overall, the paper was clearly written and thorough in its coverage. I have made a couple of general comments and a series of specific comments that I hope are useful to consider.

 Best wishes,

 Nic Badcock

**General comments**

1. EEG + Data Quality
  It’s useful to know that the external noise doesn’t affect the looking time, but I wondered whether you could any way you could report (a) a similar assessment/estimate of disruption to the EEG and (b), more generally, reporting on data quality under these conditions (i.e., generally/overall and with respect to external noise). This may feel like a separate story, in which case, you might consider broadening the paper to include it, or narrowing the scope to just be about community acceptance (i.e., removing the element on external noise and looking time). I do believe this is useful to report, but feels like this could be expanded on to give a broader description.
2. Baseline acceptability?
  I wondered what our reference point or benchmark should be in terms of acceptability. That is, how do we know 92% high satisfaction with the testing methods and 77% high satisfaction with the visit as a whole is good/acceptable? We have thresholds for some quality indices (e.g., .7 for reliability), so I was just wondering whether there’s a point of satisfaction or refusal that would lead us to conclude that this isn’t a viable enterprise. To be sure, I don’t feel that not having this is a major issue for the paper, but it might be nice to draw on previous literature or, if it doesn’t exist, to start this discussion. Maybe there’s equivalent acceptability data in high-income communities which could be considered a control of sorts.
3. An information-consent idea
  Some friends/colleagues of mine are involved in work in South Africa and have been entertaining the idea of creating graphical information and consent forms, inspired by https://www.comicbookcontracts.com/. Given some of the communication difficulties/misunderstandings that came up in the current research, I wondered whether something like this might be worth pursuing. This isn’t something that has a bearing on the current paper but felt like a good opportunity to share the idea. Very happy to put you in touch with the people thinking about this if that’s helpful.

**Specific comments:**

1. Page 1: Abstract: Results. As is, the Results are quite descriptive. This isn’t a problem per se, but I wondered if more emphasis could be put on the findings. It’s useful to have the themes reported, but, provided there’s space, it feels like it would be more informative to describe what was found for each of the themes. Relatedly, including some of the quantitative findings (e.g., relationship between noise and looking times) feels like it would provide a more thorough covering of what was found.
2. Page 3 “ *…may become apparent (e.g. for review: 16...*” > missing parenthesis at the end of the sentence here
3. Page 3: “ *…have been no published reports of how acceptable EEG technology is to parents and children within communities…*” > Although it doesn’t include eye-tracking, I think it might be worth mention Bhavnani et al. (2022) in this context.
4. Page 5 “ *…EEG cap on the child’s head the child…*” > seems to be repetition of ‘ *the child*’ here – please check
5. Page 5: “ *…Hindi nursery rhyme used (average score for familiarity: 4.2/5).*” > it would be useful to report the details of the familiarity scale
6. Page 7: “ *No significant effect was observed (F(2, 31) = .11, p = .90).*” > Best to include the effect size for all inferential comparisons

**Reference**

 Bhavnani, S., Parameshwaran, D., Sharma, K. K., Mukherjee, D., Divan, G., Patel, V., & Thiagarajan, T. C. (2022). The Acceptability, Feasibility, and Utility of Portable Electroencephalography to Study Resting-State Neurophysiology in Rural Communities. *Frontiers in Human Neuroscience*, *16*. https://www.frontiersin.org/articles/10.3389/fnhum.2022.802764

Is the work clearly and accurately presented and does it cite the current literature?

Yes

If applicable, is the statistical analysis and its interpretation appropriate?

Yes

Are all the source data underlying the results available to ensure full reproducibility?

Yes

Is the study design appropriate and is the work technically sound?

Yes

Are the conclusions drawn adequately supported by the results?

Yes

Are sufficient details of methods and analysis provided to allow replication by others?

Yes

Reviewer Expertise:

I'm a cognitive neuroscientist with a background in psychology and quantitative methods. I have some experience with qualitative methods.

I confirm that I have read this submission and believe that I have an appropriate level of expertise to confirm that it is of an acceptable scientific standard, however I have significant reservations, as outlined above.

## References

[1] : The Acceptability, Feasibility, and Utility of Portable Electroencephalography to Study Resting-State Neurophysiology in Rural Communities. *Front Hum Neurosci* .2022;16: 10.3389/fnhum.2022.802764 802764 10.3389/fnhum.2022.802764 35386581PMC8978891

# Response

**Published:** 2023-10-06

**Authors:** Georgia Lockwood Estrin

**Affiliations:** Birkbeck University of London, UK

**We thank you for your insightful comments. Please see our responses point for point outlined below:**

 1. EEG + Data Quality

 It’s useful to know that the external noise doesn’t affect the looking time, but I wondered whether you could any way you could report (a) a similar assessment/estimate of disruption to the EEG and (b), more generally, reporting on data quality under these conditions (i.e., generally/overall and with respect to external noise). This may feel like a separate story, in which case, you might consider broadening the paper to include it, or narrowing the scope to just be about community acceptance (i.e., removing the element on external noise and looking time). I do believe this is useful to report, but feels like this could be expanded on to give a broader description.

**We agree with this comment, however this is out of the scope of this paper. However, authors will be conducting a future analysis that includes this assessment of EEG disruption as part of a different paper that brings together EEG data collected in multiple settings. We have therefore added a sentence to highlight the importance of future work in this area in our Discussion section, which reads: “A future crucial next step is to evaluate the quality of EEG data collected in community settings across multiple countries to assess its feasibility for use at scale by non-experts.” Furthermore, please see our response to Reviewer 1 above regarding EEG quality, and our addition of Figure 1 to the manuscript which demonstrates a grand averaged plot from a sub-sample of participants.**

 2. Baseline acceptability?

 I wondered what our reference point or benchmark should be in terms of acceptability. That is, how do we know 92% high satisfaction with the testing methods and 77% high satisfaction with the visit as a whole is good/acceptable? We have thresholds for some quality indices (e.g., .7 for reliability), so I was just wondering whether there’s a point of satisfaction or refusal that would lead us to conclude that this isn’t a viable enterprise. To be sure, I don’t feel that not having this is a major issue for the paper, but it might be nice to draw on previous literature or, if it doesn’t exist, to start this discussion. Maybe there’s equivalent acceptability data in high-income communities which could be considered a control of sorts.

**We thank the reviewer for this comment. To our knowledge there is not an equivalent for acceptability data, but this is an interesting point for future investigation. We have therefore added a sentence in our discussion, which reads: “One important consideration is that there is currently no specific threshold set for acceptability of child development assessments – future work is needed to set such standards, which can be utilised for large scale studies; this is particularly important for monitoring data collection if such assessments are to be used at scale.”**

 3. An information-consent idea

 Some friends/colleagues of mine are involved in work in South Africa and have been entertaining the idea of creating graphical information and consent forms, inspired by https://www.comicbookcontracts.com/. Given some of the communication difficulties/misunderstandings that came up in the current research, I wondered whether something like this might be worth pursuing. This isn’t something that has a bearing on the current paper but felt like a good opportunity to share the idea. Very happy to put you in touch with the people thinking about this if that’s helpful.

**Thank you very much for this suggestion – we would be delighted to be put in touch with this group.** 

 Specific comments:

 1.Page 1: Abstract: Results. As is, the Results are quite descriptive. This isn’t a problem per se, but I wondered if more emphasis could be put on the findings. It’s useful to have the themes reported, but, provided there’s space, it feels like it would be more informative to describe what was found for each of the themes. Relatedly, including some of the quantitative findings (e.g., relationship between noise and looking times) feels like it would provide a more thorough covering of what was found.

**Thank you for highlighting this – we have added three additional sentences to the Results section of the Abstract, including: “A high level of child engagement in EEG tasks was demonstrated, with children’s gaze at the screen during the task averaging at 85.4% (±12.06%) of the task time. External distractions and noise during the tasks were measured, but not found to significantly effect child’s attention to the screen during EEG tasks”; and “Results from interviews and the survey indicated acceptability for gaze-controlled EEG research for parents and children”; and “Barriers to participation mainly included logistical challenges, such as geographic location and time, whereas involvement of the wider family in decision making was highlighted as an important facilitator to partake in the research.”**

 2. Page 3 “…may become apparent (e.g. for review: 16...” > missing parenthesis at the end of the sentence here

**Thank you for highlighting this – we have added the missing parenthesis**

 3. Page 3: “…have been no published reports of how acceptable EEG technology is to parents and children within communities…” > Although it doesn’t include eye-tracking, I think it might be worth mention Bhavnani et al. (2022) in this context.

**We have included the following sentence in this section: “Whilst one paper has used EEG in a large population-based study conducted in rural settings in India 26, no qualitative work was conducted to understand the acceptability of the technology to parents and children within these communities.”**

 4. Page 5 “…EEG cap on the child’s head the child…” > seems to be repetition of ‘the child’ here – please check

**Thank you for pointing this out – we have adapted**

 5. Page 5: “…Hindi nursery rhyme used (average score for familiarity: 4.2/5).” > it would be useful to report the details of the familiarity scale

**We have added the following information: “(average score for familiarity: 4.2 score out of a 0 to 5 point Likert scale of reporting on familiarity).”**

 6.    Page 7: “No significant effect was observed (F(2, 31) = .11, p = .90).” > Best to include the effect size for all inferential comparisons

**Thank you for this comment - in line with our previous work and statistical reporting recommendations for psychology papers, we remove statistical results where no significant effect was observed.**

# Reviewer report: Reviewer response for version 1

**DOI:** 10.21956/wellcomeopenres.19164.r49312

**Published:** 2022-05-03

**Authors:** Wanze Xie

**Affiliations:** 1 School of Psychological and Cognitive Sciences, Peking University, Beijing, China

This manuscript pertains to a topic of importance – the acceptability of using EEG and eye-tracking measures with children living in low- and middle-income countries (LMICs). I really admire the authors’ sheer effort to collect neurophysiological data and conduct neurodevelopment assessments in such low-resource community settings in India. In this manuscript, the authors provide useful information with informative tables (e.g., Table 3 and 4) summarizing the barriers and facilitators for the locals to participate in such a comprehensive cognitive assessment battery (Braintools). The manuscript is already well-written. I only have three comments/questions that I would like the authors to address in a revision.

1. Please provide more detailed information about the Braintools battery. I understand a paper has been published describing this battery (Haartsen *et al.*,2021), but I think more information about it would be much appreciated by the readers, such as the details on the EEG/behavioral tasks included and the gaze-contingent stimulus presentation approach.
2. I personally found that eye-tracking calibration for young children can be a big issue for gaze-contingent paradigms and simultaneous EEG and eye-tracking data collection and analysis, not to mention that my experiences are limited to lab settings. For example, our Tobii eye-tracker might lose the track of the child’s fixation if they move drastically, and the process of regaining their gaze and re-calibrating is complex. Thus, I wonder if the authors have had similar issues in using their gaze-contingent stimulus presentation paradigm in community settings, and how they have resolved the issues.
3. I would love to see some sample EEG and eye-tracking data analyzed and plotted in the manuscript, such as a PSD plot, a grand average ERP plot, and a figure illustrating children’s looking behaviors and reaction time. It is important to know whether the quality of the data collected in community settings in LMICs is acceptable.

Is the work clearly and accurately presented and does it cite the current literature?

Yes

If applicable, is the statistical analysis and its interpretation appropriate?

Yes

Are all the source data underlying the results available to ensure full reproducibility?

Partly

Is the study design appropriate and is the work technically sound?

Yes

Are the conclusions drawn adequately supported by the results?

Yes

Are sufficient details of methods and analysis provided to allow replication by others?

Partly

Reviewer Expertise:

EEG/ERP, child development

I confirm that I have read this submission and believe that I have an appropriate level of expertise to confirm that it is of an acceptable scientific standard, however I have significant reservations, as outlined above.

# Response

**Published:** 2023-10-06

**Authors:** Georgia Lockwood Estrin

**Affiliations:** Birkbeck University of London, UK

Thank you for your excellent comments. We have responded to each in line below:

 1. Please provide more detailed information about the Braintools battery. I understand a paper has been published describing this battery (Haartsen et al.,2021), but I think more information about it would be much appreciated by the readers, such as the details on the EEG/behavioral tasks included and the gaze-contingent stimulus presentation approach.

**Thank you for this comment. We have included some additional details about the battery, and this section now reads: “The complete Braintools battery lasted approximately 35 minutes, and included a mixture of static pictures presented when the child looked at the screen (e.g. faces, objects, checkerboards), dynamic videos (nursery rhymes sung in Hindi or toys moving – each video was approximately one-minute long) or sounds while the child either played with toys or watched a cartoon. For more information on the Braintools eye-tracking and EEG battery, see 23, but in summary the Braintools battery included first the FastERP task, interspersed with short cartoon clips and dynamic videos, followed by an auditory oddball task (see 23 Figure 1 for additional detail on tasks).”**

 2. I personally found that eye-tracking calibration for young children can be a big issue for gaze-contingent paradigms and simultaneous EEG and eye-tracking data collection and analysis, not to mention that my experiences are limited to lab settings. For example, our Tobii eye-tracker might lose the track of the child’s fixation if they move drastically, and the process of regaining their gaze and re-calibrating is complex. Thus, I wonder if the authors have had similar issues in using their gaze-contingent stimulus presentation paradigm in community settings, and how they have resolved the issues.

**We thank the reviewer for highlighting this challenge – which is an important consideration when working with young children and can impact eye-tracking data collection. We found similar challenges in community settings, and these were resolved by providing breaks to the children and recalibrating the eye-tracker when needed. To highlight this, we have added the following sentence into our “Challenges in data collection” section of the Results: “Child’s inattention or distraction resulted in needing to take breaks and needing to recalibrate the eye-tracker if the child moved; this impacted the time of the assessment overall.”**

 I would love to see some sample EEG and eye-tracking data analyzed and plotted in the manuscript, such as a PSD plot, a grand average ERP plot, and a figure illustrating children’s looking behaviors and reaction time. It is important to know whether the quality of the data collected in community settings in LMICs is acceptable.

**Thank you for this important comment. Whilst this was not an aim of the paper, we agree that this is an important addition to show that the data was of high quality to allow for analysis in future papers. We have therefore added Figure 1 to the manuscript, which shows grand average plots of the: 1a)  power spectrum during whole session; 1c) fast ERPs; 1b) power spectra during the dynamic video task. We have also added a section in the Supplementary Material with detailed information of the analysis of these plots, as well as individual plots of participants included in this sub-analysis.**
